# Automated crack detection of train rivets using fluorescent magnetic particle inspection and instance segmentation

**DOI:** 10.1038/s41598-024-61396-6

**Published:** 2024-05-09

**Authors:** Haoguang Wang, Wangzhe Du, Guanhua Xu, Yangfan Sun, Hongyao Shen

**Affiliations:** 1 The State Key Laboratory of Fluid Power and Mechatronic Systems, College of Mechanical Engineering, Zhejiang University, Hangzhou, 310027 Zhejiang China; 2 Key Laboratory of 3D Printing Process and Equipment of Zhejiang Province, College of Mechanical Engineering, Zhejiang University, Hangzhou, 310027 Zhejiang China; 3 College of Mechanical and Vehicle Engineering, Taiyuan University of Technology, Taiyuan, 030002 Shanxi China

**Keywords:** Crack detection, Rail transit, Deep learning, Computer vision, Fluorescent magnetic particle inspection, Composite magnetization, Engineering, Mechanical engineering

## Abstract

The railway rivet is one of the most important and easily damaged parts of the connection. If rivets develop cracks during the production process, their load-bearing capacity will be reduced, thereby increasing the risk of failure. Fluorescent magnetic particle flaw detection (FMPFD) is a widely used inspection method for train fasteners. Manual inspection is not only time-consuming but also prone to miss detection, therefore intelligent detection system has important application value. However, the fluorescent crack images obtained by FMPFD present challenges for intelligent detection, such as the dense, multi-scaled and uninstantiated cracks. In addition, there is limited research on fluorescent rivet crack detection. This paper adopts instance segmentation to achieve automatic cracks detection of rivets. A decentralized target center and low overlap rate labeling method is proposed, and a Gaussian-weighted correction post-processing method is introduced to improve the recall rate in the areas of dense cracks. An efficient channel spatial attention mechanism for feature extraction is proposed in order to enhance the detection of multi-scale cracks. For uninstantiated cracks, an improvement of crack detection in uninstantiated regions based on multi task feature learning is proposed, thoroughly utilizing the semantic and spatial features of the fluorescent cracks. The experimental results show that the improved methods are better than the baseline and some cutting-edge algorithms, achieving a recall rate and mAP_0.5_ of 86.4% and 90.3%. In addition, a single coil non-contact train rivet composite magnetization device is built for rivets that can magnetize different shapes of rivets and has universality.

## Introduction

### Background

**Figure 1:**
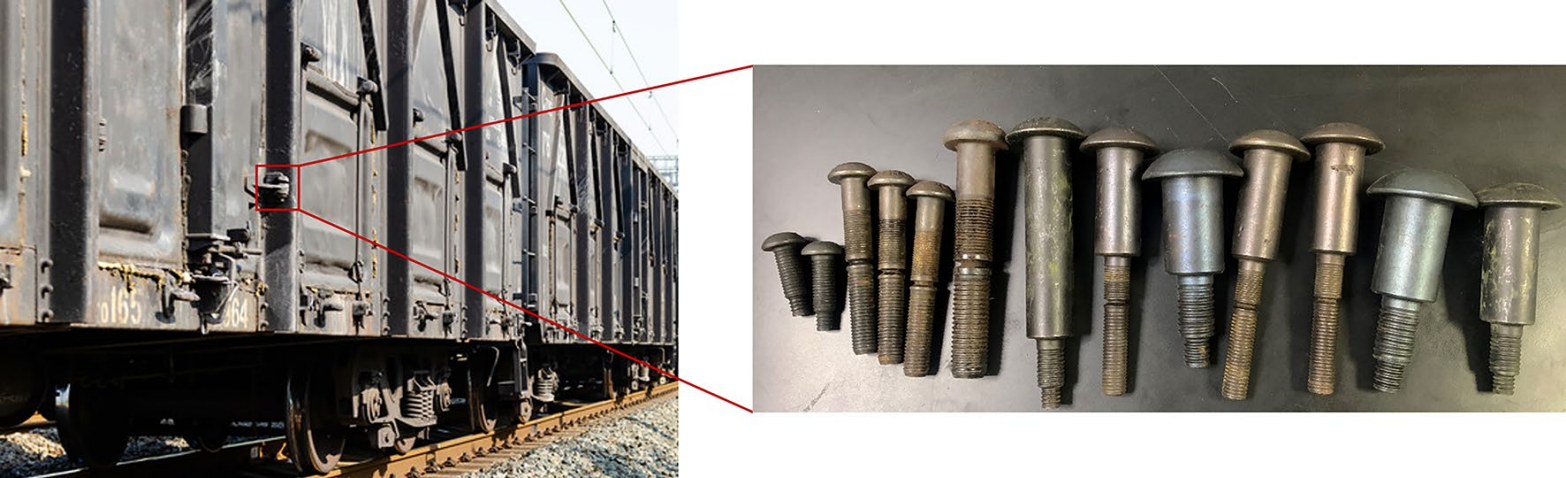
Defective rivets for train parts.

Rivets and pins are widely used for connecting parts in railway vehicles with high speed and heavy load^1^, as shown in Fig. 1. Rivets have the advantages of high fastening force and high shear resistance, which can effectively alleviate the troubles caused by vibration and swing. However, some cracks may occur at the rivet head due to technical reasons in the forging process, which reduce the product performance. These small cracks may expand under the load and stress exerted on the rivet, leading to failure. Currently, industrial fluorescent magnetic particle flaw detection (FMPFD) relies on manual observation to detect defective rivets, which is not only labor-intensive but also prone to miss inspection. In order to improve standardization and work efficiency, intelligent detection system needs to be introduced. It offers an intelligent detection solution and broad application prospect for rivet defect detection^2^.

### Principle of fluorescent magnetic particle inspection

Non-destructive testing (NDT) methods are techniques that allow the inspection of materials and components without causing any damage or altering their functionality^3^. In particular, fluorescent magnetic particle testing is a popular method in the railway manufacturing industry, as it can quickly and visually detect defects in car body welds, diesel engine parts, and steel forgings.

When an external magnetic field is applied to the workpiece, the magnetic flux lines encounter a discontinuity due to the presence of a defect. The air gap in the defect has much lower permeability than the workpiece material, so the flux lines are distorted and forced to cross the defect. As a result, some flux lines pass under the defect, while others leak out of the workpiece and re-enter at another point. These leakage flux lines form a magnetic dipole (N and S poles) on both sides of the defect. By spraying fluorescent magnetic particles on the workpiece surface, the defect can be revealed as the particles are attracted by the leakage flux. Under suitable ultraviolet light, the particles emit a visible glow that indicates the location, size and shape of the defect. The type and severity of the defect can be inferred from the pattern of the particles.

## Research status of intelligent detection of cracks and fluorescent cracks

This work aims to improve the performance of fluorescent crack detection by using a deep learning-based detection and segmentation algorithm. Traditional machine learning methods, such as support vector machines^4^, artificial neural networks^5^, decision trees^6^, and K-means clustering algorithms^7^, are widely used in the field of crack detection. However, these methods require complex image preprocessing and are sensitive to the quality of the samples, which poses a challenge for industrial product inspection.

Deep learning-based crack detection has been widely adopted in various fields, and several methods have been proposed in the literature. Li et al.^8^ integrated a deep convolutional neural network (DCNN) with a classical edge detector to reduce the noise and improve the accuracy of concrete crack identification. Yang et al.^9^ proposed a feature pyramid and enhanced layered network for pavement crack detection, which incorporated context information into low-level features and balanced the loss function using layered weights and nested samples, achieving better accuracy and generalization than existing methods. Li et al.^10^ developed a bridge crack classification model based on CNNs, which used a sliding window algorithm to segment the image and a search strategy combining the image pyramid and region of interest to speed up the process, resulting in a high recognition performance and generalization ability. Liu et al.^11^ put forward a novel method for composite thermography defect detection and quantification based on a multi-feature vision transformer model and an infrared data sampling strategy. Long et al.^12^ realized a fatigue crack growth rate measurement method based on deep learning and mobile phone, which can use crack data sets of different scales to predict crack length, and combine fracture mechanics to effectively measure small cracks, and apply to non-standard specimens. There are differences between fluorescent magnetic particle crack detection and conventional crack detection, so the deep learning algorithm needs to be improved. Deng^13^ constructed a bilinear symmetric network model, NASNet, to capture the subtle local differences between defect-free and defective images, which effectively improved the detection accuracy of fluorescent cracks on cylindrical workpieces. Xiang et al.^14^ proposed an improved detection model, AlexNet, which combined deep separable convolution to quickly and accurately identify cracks under noise interference, reflective shielding, and low-brightness conditions, with higher precision and recall. Tout et al.^15^ established an automatic visual system for crankshaft surface inspection based on magnetic particle testing technology. Yang et al.^16^ proposed a bearing ring defect recognition method based on visual features and high-level features, which could extract crack defects in complex texture backgrounds better than traditional methods.

Object detection is a research area that mainly focuses on natural scenes and face detection^3^. However, there is a lack of research on rivet fluorescent crack detection, which is an important task in manufacturing inspection. Rivet fluorescent crack images have distinctive features that make them challenging to detect, such as dense cracks, irregular shapes, and multi-scale variations. Moreover, the non-uniform spraying of fluorescent magnetic particles causes fluorescent aggregation, which obscures the cracks and hinders defect identification.

### Main content

In this paper, instance segmentation is used to achieve automatic cracks detection of rivets. To solve the problem of low precision in fluorescent crack detection due to dense, multi-scale, and uninstantiated cracks, this paper proposes a decentralized target center and low overlap rate labeling method, and introduces a Gaussian-weighted correction post-processing method for improving recall rate in dense regions, an efficient channel spatial attention mechanism (ECSAM) adaptively fuse different feature maps and multi task feature learning. The rest of this paper is organized as follows: the second section introduces the overall process of fluorescent magnetic particle inspection for rivets and the single coil non-contact train rivet composite magnetization device. The third section shows the fabrication of the fluorescence crack dataset. The fourth section introduces the improved algorithms and analyses the reasons for its effectiveness. In the fifth section, experiments are designed to evaluate the performance of the proposed methods. The sixth section gives the conclusion and discussion of this work.

## A single coil non-contact train rivet composite magnetization device

### Overall process of fluorescent magnetic particle inspection for rivets

**Figure 2:**
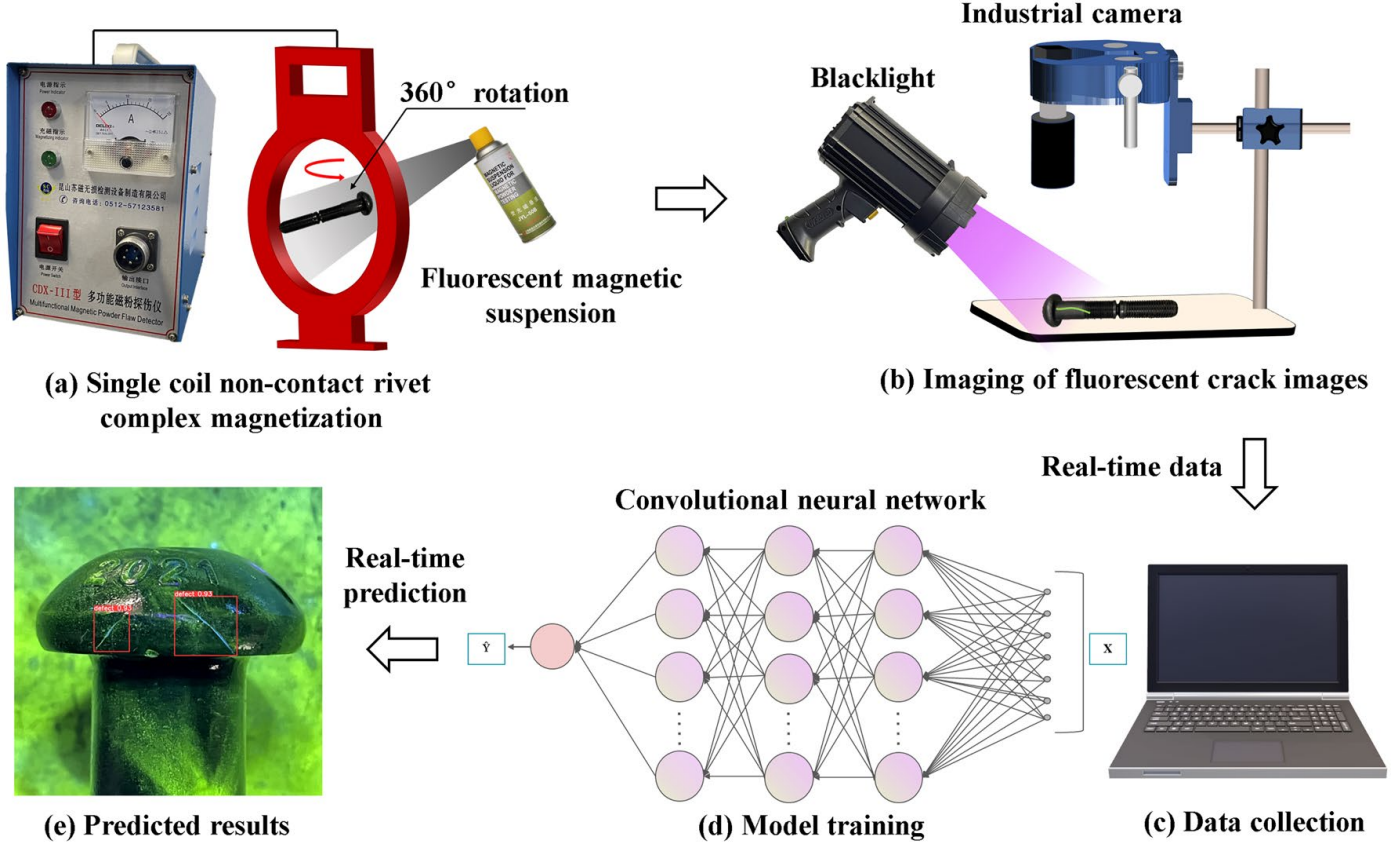
Overall process of fluorescent magnetic particle inspection for rivets.

A single coil non-contact train rivet composite magnetization device is built, which is equipped with an O-type magnetic field distribution regulator that could achieve composite magnetization of rivets. The device works by applying a fixed magnetic field along the coil axis to the rivet, which is placed at the center of the O-type regulator where the magnetic intensity is the highest. The fluorescent magnetic particles are applied uniformly as the rivet is rotated. This is equivalent to applying a synthetic rotating magnetic field to the stationary rivet. The composite magnetization allows simultaneous detection of both axial and radial cracks of the rivet. Figure 2 illustrates the specific implementation process of the device. The conventional equipment used in the industry is more suitable for large parts. It also requires direct contact and axial current transmission to detect radial cracks in rivets, which can cause sparks that burn the rivet surface and interfere with the observation of fluorescent cracks.

The proposed method achieves composite magnetization by using the magnetic field, instead of applying current to the rivets. This method has several advantages, such as being non-contact, suitable for small parts, and insensitive to the shape of the parts. Therefore, the device can be generalized to the composite magnetization of parts with various shapes.

### Introduction of the single coil non-contact train rivet composite magnetization device

**Figure 3:**
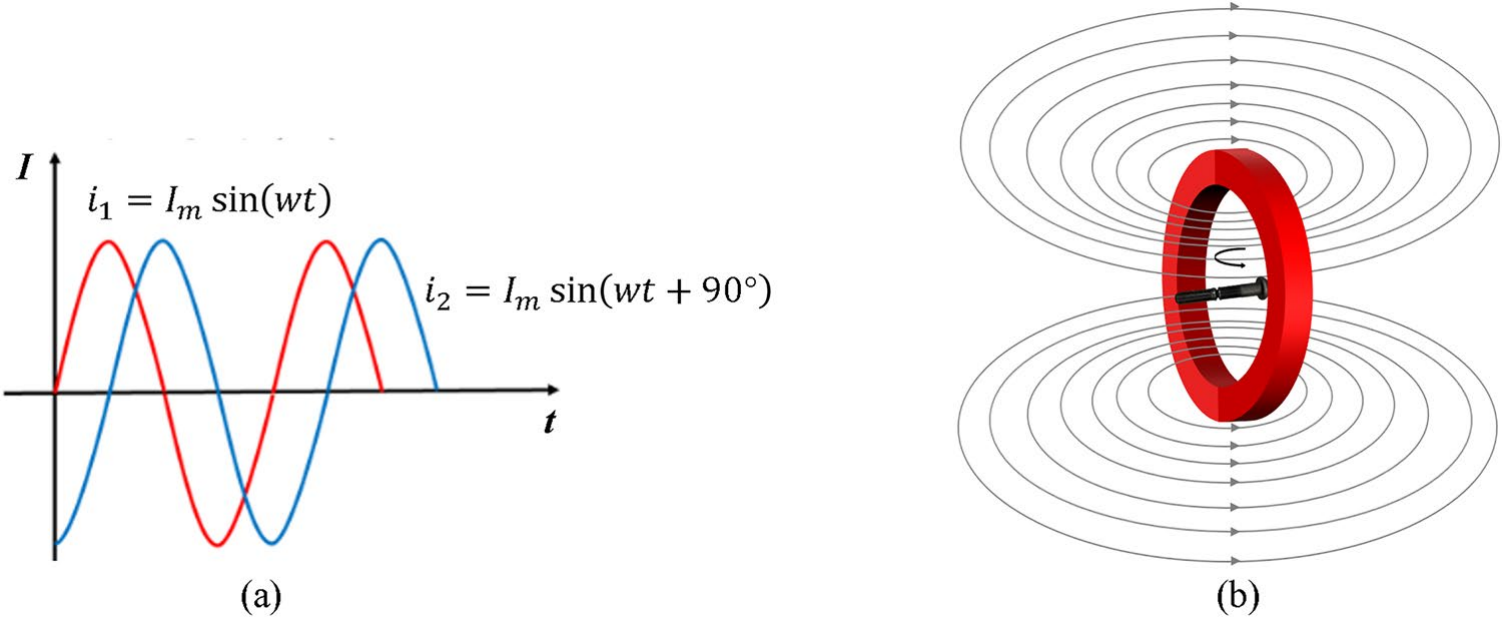
Principle of composite magnetization with (**a**) Composite magnetic field formed by current induction with a phase difference, (**b**) Magnetic field distribution of O-type magnetic field regulator.

Industrial production often relies on composite magnetization equipment, which is bulky and costly. In this work, a single coil non-contact train rivet composite magnetization device is built that can achieve the same effect. The device consists of two main components: a magnetic field generator and an O-type magnetic field distribution regulator. It is easy to operate and carry, making it suitable for various inspection scenarios. Figure 3b illustrates the magnetic field distribution of the O-type regulator. Table 1 shows the parameters of the devices used for the fluorescent magnetic particle inspection of rivets.

**Table 1:** Parameters of devices for composite magnetization.

Parameters	Values
Input voltage	220 V
Output current	15A
Regulator type	Annular regulator (O type)
Regulator inner diameter	150 mm
Central magnetic field intensity	≥ 1500e
Blacklight irradiance	More than 8000 $\upmu {\text{W}}/{\text{cm}}^{2}$ at 381 mm
Magnetic suspension concentration	0.5 mL$/$100 mL

### Principle of composite magnetization

A widely used magnetization method in industrial production is compound magnetization, which combines longitudinal and circumferential magnetization. This method creates a longitudinal magnetic field along the workpiece axis and a closed circumferential magnetic field around the workpiece, perpendicular to the axis. This way, both radial and axial defects of the workpiece can be detected simultaneously. Compound magnetization is achieved by applying a synthetic magnetic field to the workpiece, which is generated by two single-phase sinusoidal AC currents with a certain phase difference. Figure 3a illustrates the principle of the synthetic magnetic field.

1 $${i}_{1}={I}_{m}{\text{sin}}\left(wt\right)$$

2 $${i}_{2}={I}_{m}{\text{sin}}(wt+90^\circ )$$

3 $${i}_{1}={I}_{m}\angle 0^\circ$$

4 $${i}_{2}={I}_{m}\angle 90^\circ$$

${i}_{1}$ and ${i}_{2}$ are two alternating currents with the same amplitude ${I}_{m}$ and a phase difference of 90°. $wt$ is the angular frequency. ${\text{sin}}\left(wt\right)$ and ${\text{sin}}(wt+90^\circ )$ are two sinusoidal functions, which describe the waveforms of the alternating currents. ${I}_{m}\angle 0^\circ$ and ${I}_{m}\angle 90^\circ$ represent the vector forms of the alternating currents. These two sinusoidal alternating currents with phase difference can be regarded as the projections of two rotating vectors, which rotate in different directions with the same angular velocity on the complex plane. When the resultant vector of these two rotating vectors rotates in space, it generates a rotating magnetic field, whose magnetic field strength and direction are determined by the modulus and polar angle of the resultant vector.

### Composite magnetization validation

**Figure 4:**
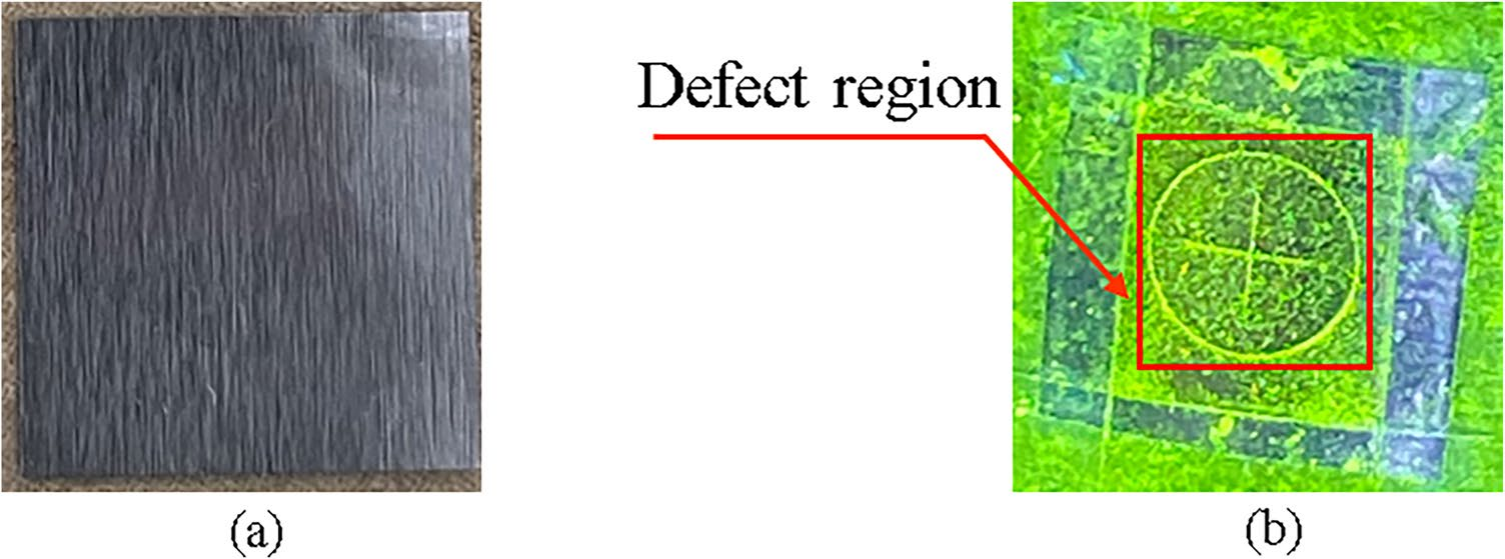
Experimental verification of composite magnetization effect with (**a**) Test piece before composite magnetization, (**b**) “Cross” fluorescent magnetic trace.

An experiment is conducted to further verify the composite magnetization effect. A 50 µm thick D-type magnetic sensitivity test piece is attached to the surface of the rivet. The test piece is engraved with a 30 µm deep "cross" crack. The cracked side of the test piece adheres closely to the surface of the rivet. This simulated a crack that is 20 µm below the surface of the test piece. The rivet is magnetized and rotated at the center of the O-type regulator for several seconds, while being sprayed with fluorescent magnetic suspension continuously. The defects caused magnetic leakage, and the fluorescent magnetic particles accumulated near the defects. The test piece displayed a “cross” fluorescent magnetic trace. Figure 4 demonstrates that our method can detect both radial and axial defects of the rivet, which confirms that the method can achieve composite magnetization.

## Datasets

The images of rivets with cracks, each with a resolution of 1500 × 1500 pixels, are collected from industrial production. The images captured from different riveted structure surfaces were preprocessed to unify their resolution to 1500 × 1500 pixels. This can ensure the quality and consistency of the images, as well as avoid the loss of defect details or the reduction of detection generalization ability caused by too small or too large resolution. Moreover, such resolution is also conducive to control the size and computational cost of the model, and to improve the precision and recall of the detection. The rivets were formed by warm forging technology. This technique involves heating the metal below the recrystallization temperature but above the blue brittleness temperature, where the metal has low tensile strength and high plasticity. Then, a certain pressure is applied to the metal blank to make it deform plastically in the mold cavity, producing a rivet with a desired shape and size. However, this process causes stress deformation in the rivet head, which makes the rivet susceptible to longitudinal and transverse forces during stamping. As a result, some rivets develop hidden cracks.

**Figure 5:**
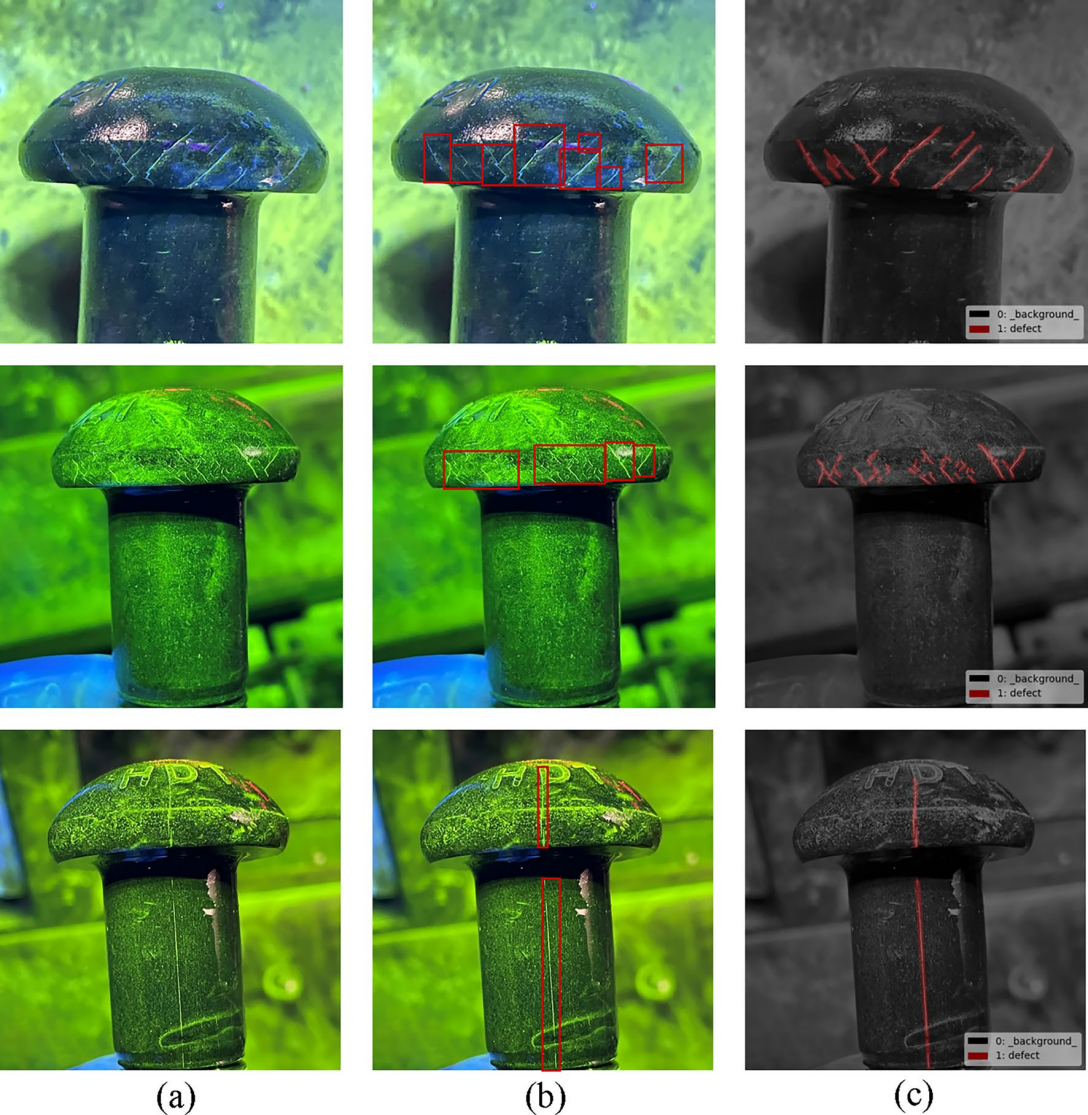
Examples of annotation results with (**a**) Images, (**b**) Object detection annotation, (**c**) Segmentation annotation.

Figure 5 shows that the cracks are mainly located at the rivet head, which are dense, irregular and multi-scale. Most of the strip cracks can be considered as distinct targets. However, some small cracks are distributed near the strip cracks, which are difficult to be considered as individual targets, compare to the stripe crack. Moreover, some cracks spread across the rivet head. The long cracks penetrate both the rivet head and body, which results in a large variation in crack scales.

In this work, the fluorescent cracks in the rivet images are focused on. The corresponding annotation tools are used to complete the segmentation and detection annotations. The category and contour coordinates of each defect are saved in the corresponding JASON file by the segmentation tool, and the overall defect targets are saved in the corresponding XML file by the annotation tool, following the formats of COCO and VOC datasets. Typical defects are extracted from the original dataset. The corresponding annotations are displayed in Fig. 5.

## Research on key algorithms

**Figure 6:**
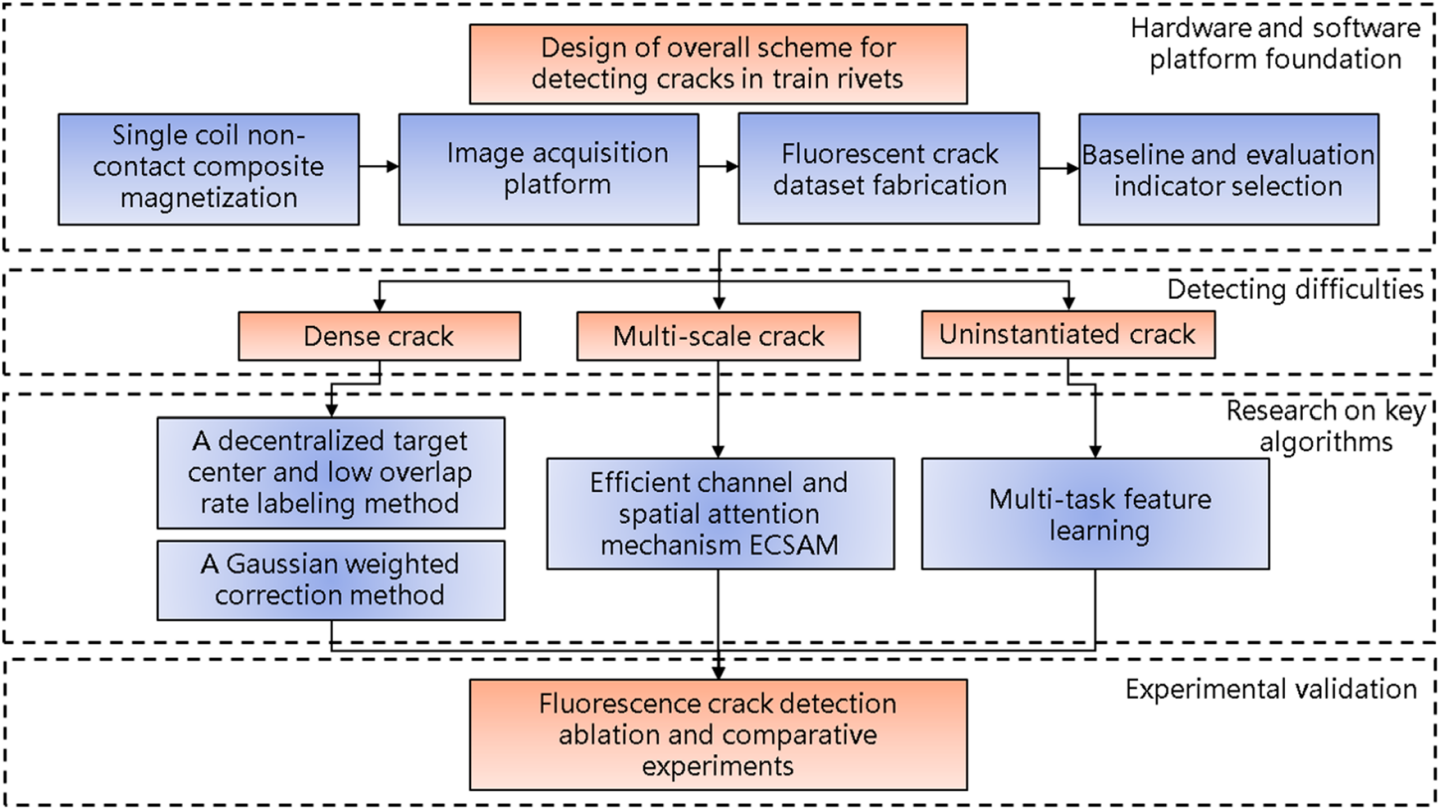
Overall architecture.

Researchers have introduced deep learning into the field of rivet crack detection to improve its precision and robustness in complex scenes. YOLOv5, a classical deep learning-based object detection algorithm, can achieve high speed and accuracy, which makes it suitable for industrial applications. However, according to the analysis of the fluorescent cracks, YOLOv5 still has some problems with missing and false detections. Therefore, the recall rate and mAP of the algorithm need to be further improved. The overall architecture is shown in Fig. 6.

### Research on crack detection in the dense area

#### Design of decentralized target center and low overlap rate labeling method

YOLOv5 uses a grid-based approach to predict bounding boxes for detected objects. Each grid cell corresponds to the center point of an anchor box, and the offset from the upper left corner coordinate of the grid cell is used to regress the bounding box location. It has made some improvements over previous versions^17,18^, such as using a compound scaling method and adding a new loss function. However, it still faces some challenges in annotating and detecting objects in dense crack areas, which are common in the fluorescent crack dataset.

**Figure 7:**
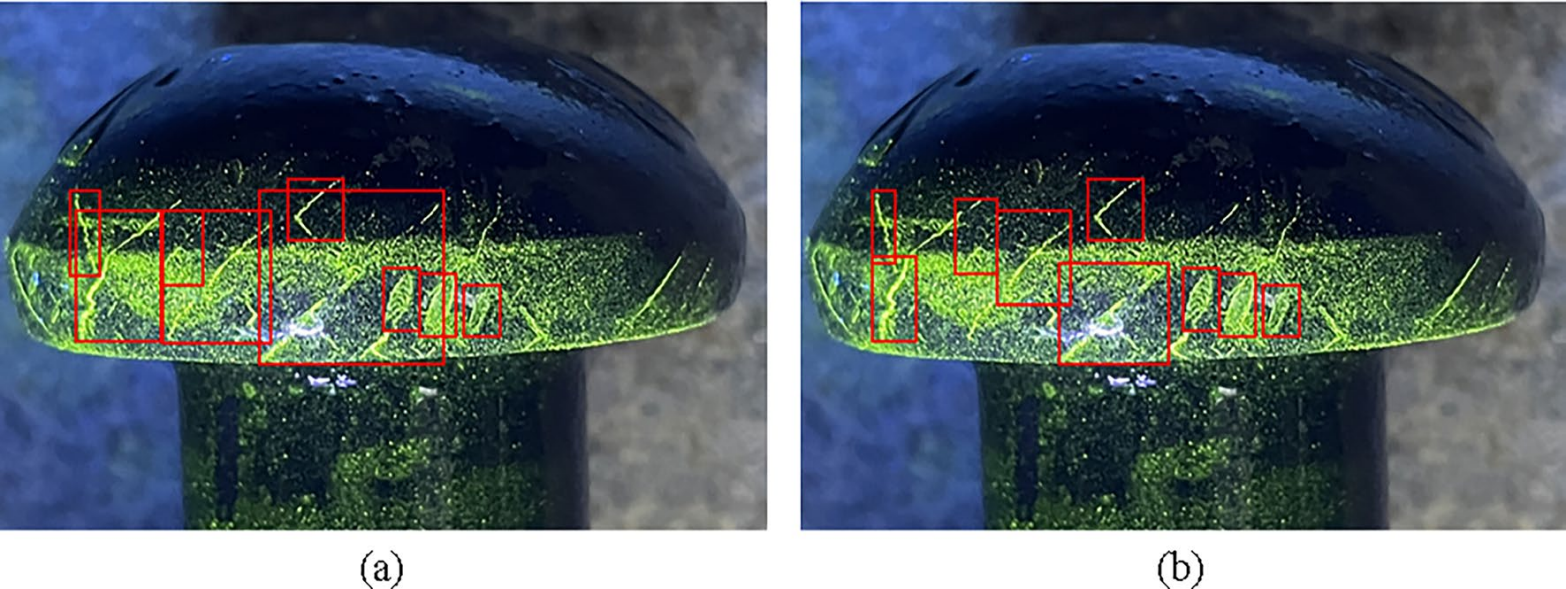
Comparison of traditional and proposed annotation methods with (**a**) Traditional annotation method, (**b**) Decentralized target center and low overlap rate labeling method.

The conventional annotation method is to draw the bounding box as close to the object as possible, but this may cause excessive overlap or inclusion between two adjacent bounding boxes, leading to missed detection or false positives. To address this problem, a decentralized target center and low overlap rate labeling method for dense crack areas is proposed, which focuses on the non-overlapping parts of the cracks and minimizes or eliminates the overlap between different bounding boxes. The decentralized target center and low overlap rate labeling method is illustrated in Fig. 7.

**Figure 8:**
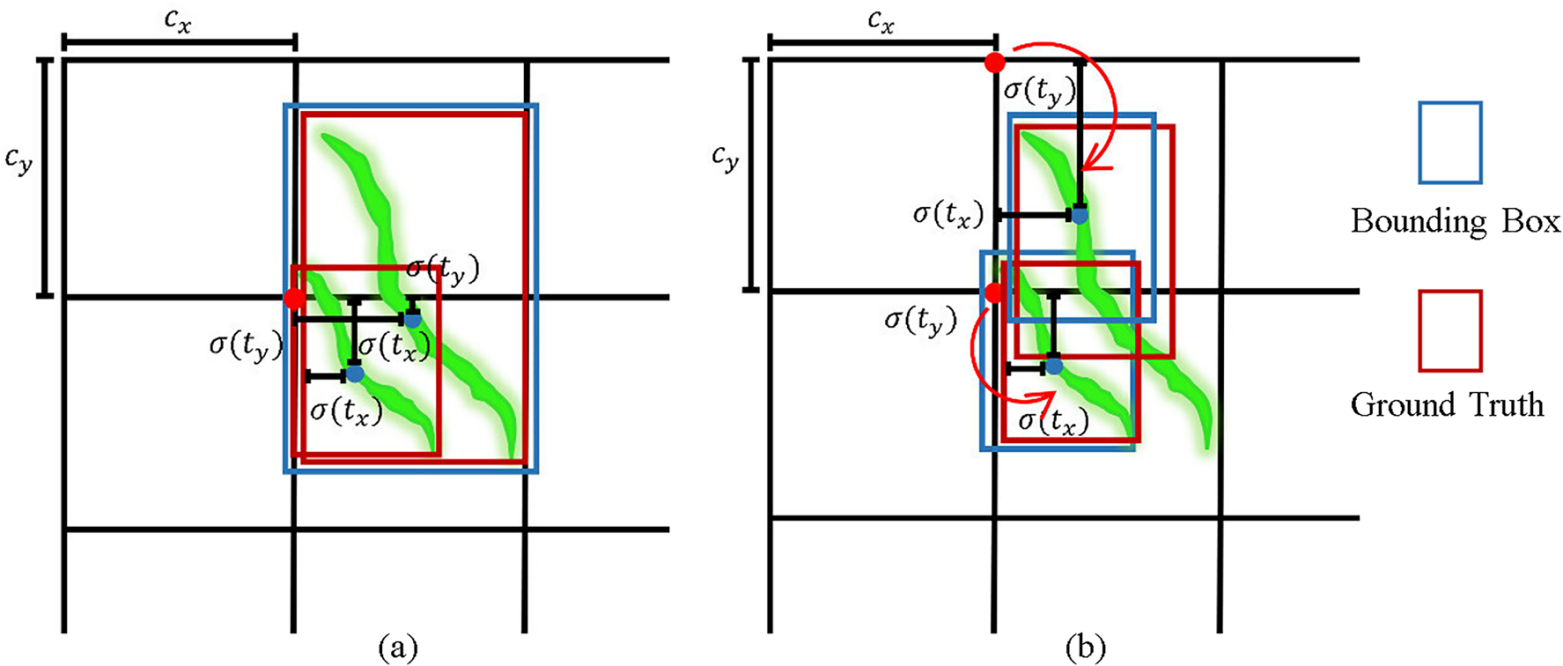
Improvement of bounding box generation by the proposed annotation method with (**a**) Traditional method, (**b**) Improved method.

In this way, the center distance between adjacent boxes can be increased while retaining the most significant features of the crack. As a result, the dense cracks are distributed to different grid cells for better prediction and regression, as shown in Fig. 8.

5 $${b}_{x}=2\sigma \left({t}_{x}\right)-0.5+{C}_{x}$$

6 $${b}_{y}=2\sigma \left({t}_{y}\right)-0.5+{C}_{y}$$

7 $${b}_{w}={P}_{w}{(2\sigma \left({t}_{h}\right))}^{2}$$

8 $${b}_{h}={P}_{h}{(2\sigma \left({t}_{w}\right))}^{2}$$

where ${C}_{x}$ and ${C}_{y}$ denote the distance of that grid from the top-left corner of the feature map. ${P}_{w}$ and ${P}_{h}$ denote the width and height of the prior box. ${b}_{x}$ and ${b}_{y}$ represent the center coordinates of the predicted bounding box. ${b}_{w}$ and ${b}_{h}$ represent the width and height of the predicted bounding box. ${t}_{x}$ and ${t}_{y}$ represent the offset of the predicted box center relative to the prior box center. ${t}_{w}$ and ${t}_{h}$ represent the scaling factors of the predicted box width and height relative to the prior box. The symbol $\sigma$ represents the sigmoid function.

**Figure 9:**
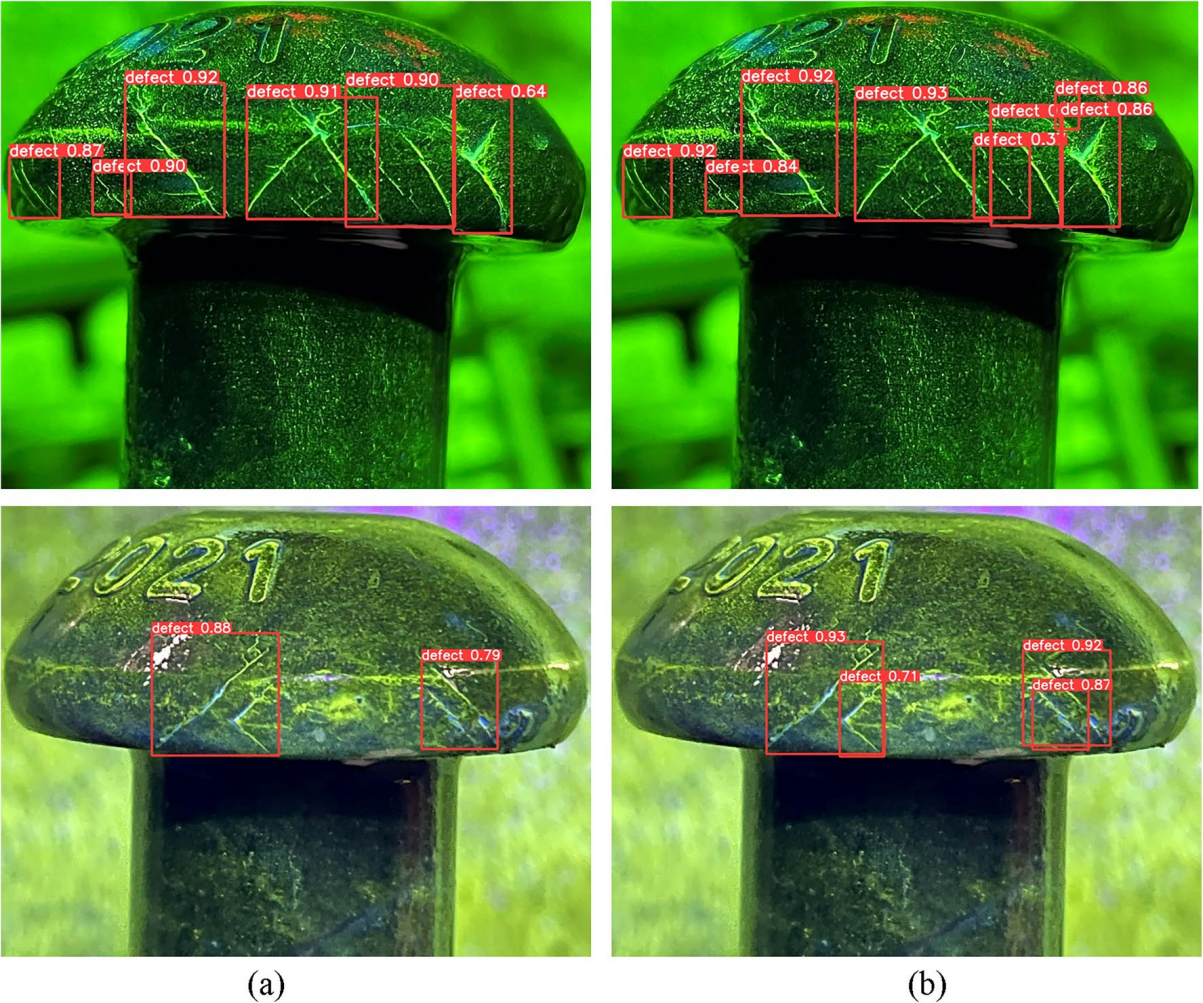
Detection examples of design of decentralized target center and low overlap rate labeling method with (**a**) YOLOv5, (**b**) YOLOv5 + Decentralized target center and low overlap rate labeling method.

To verify the effectiveness of the new annotation method, some detection results are presented in Fig. 9 and compare them with the baseline results. In YOLOv5 algorithm, the percentage above the predicted bounding box represents confidence. It indicates how certain the model is that the predicted bounding box contains the target object and reflects the accuracy of this prediction. When the bounding box accurately contains the target, the confidence is higher. As the results, our method can detect more cracks and reduce false positives in dense crack areas.

#### Gaussian-weighted correction post-processing method for improving high recall in dense regions

NMS is a post-processing technique that is commonly used in object detection tasks to eliminate duplicate detections and select the most relevant bounding boxes that correspond to the detected objects. NMS works by ranking the bounding boxes according to their confidence scores and choosing the one with the highest score as the final output. Then, it discards any remaining bounding boxes that have a high overlap (measured by intersection over union, IoU) with the chosen box and exceed a specified threshold ${N}_{t}$. However, NMS may also delete some bounding boxes that are close to the chosen box but belong to different objects, especially in dense crack areas. Therefore, NMS needs to balance between reducing redundancy and preserving diversity. NMS can be formulated as an equation at the stage of bounding box selection:

9 $${s}_{i}=\left\{\begin{array}{c}{s}_{i}, IoU\left(M,{b}_{i}\right)<{N}_{t}\\ 0, IoU\left(M,{b}_{i}\right) \ge {N}_{t}\end{array}\right.$$

In the formula, $M$ represents the selected bounding box with the highest confidence, ${N}_{t}$ is the threshold specified in the NMS, ${b}_{i}$ represents the remaining bounding boxes, ${s}_{i}$ represents the classification confidence of the corresponding bounding boxes.$IoU(\bullet )$ represents the formula for calculating the intersection over the union between the two bounding boxes. $\sigma$ is the variance of a Gaussian distribution. In this paper, $\sigma$ is set to 0.5.

NMS works by applying fixed criteria to select only one bounding box for each object. However, this may cause missed detection. To address this issue, Soft-NMS^19^ proposes to reduce the confidence of bounding boxes that are adjacent to the selected bounding box $M$, instead of removing them completely. The confidence reduction is based on a Gaussian function that depends on the IoU between the bounding boxes and the selected box. If the IoU is greater than a specified threshold ${N}_{t}$, the confidence of the adjacent bounding boxes will decay gradually, which allows more boxes to be retained and improves the recall rate.

The confidence reduction also considers the distance between the bounding boxes and the selected box $M$. The closer the bounding box is to $M$, the more its confidence will be attenuated. The original confidence will be preserved if the bounding box is far from $M$. The calculation formula of Soft-NMS is as follows:

10 $$s_{i} = s_{i} e^{{ - \frac{{ iou\left( {M,b_{i} } \right)^{2} }}{\sigma }}} , \forall b_{i} \notin D$$

**Figure 10:**
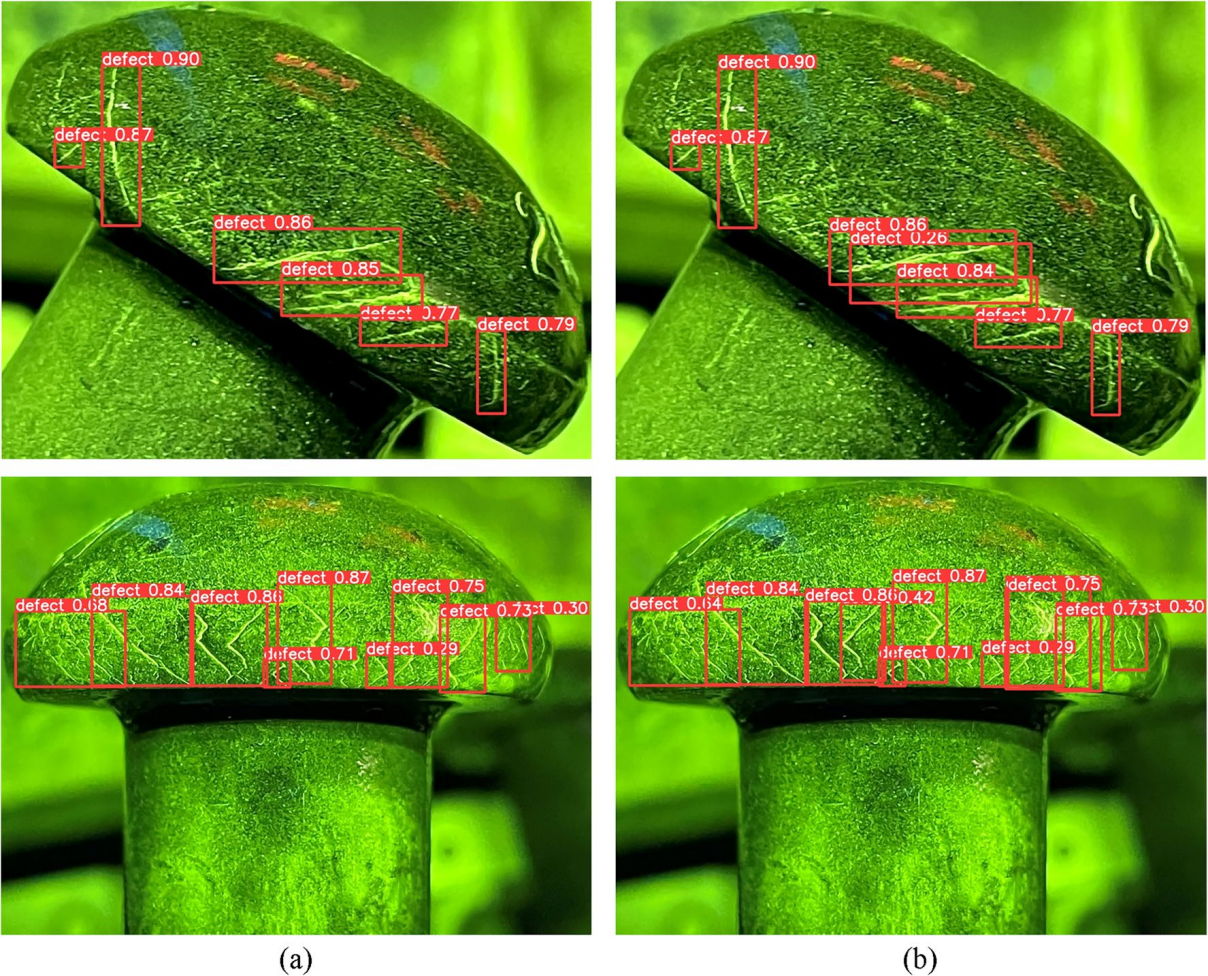
Examples of improving detection results in dense crack areas with (**a**) YOLOv5, (**b**) YOLOv5 + Gaussian-weighted correction post-processing method.

YOLOv5 is a one-stage algorithm that generates nine anchors for each grid in the prediction layer. However, this approach may cause missed detection and a low recall rate in areas with dense cracks, where multiple cracks may fall into the same grid. Moreover, if the traditional NMS method is used to select only one bounding box with the highest confidence and discard the others that overlap with it, the recall rate will further decrease. To alleviate this issue, Soft-NMS is introduced to YOLOv5 instead of NMS. Soft-NMS reduces the confidence of overlapping bounding boxes rather than eliminating them, which preserves more diversity and improves the recall rate. Figure 10 shows some detection results of the Gaussian-weighted correction post-processing method in dense crack areas.

### Improvement of feature extraction method based on efficient channel spatial attention mechanism

**Figure 11:**
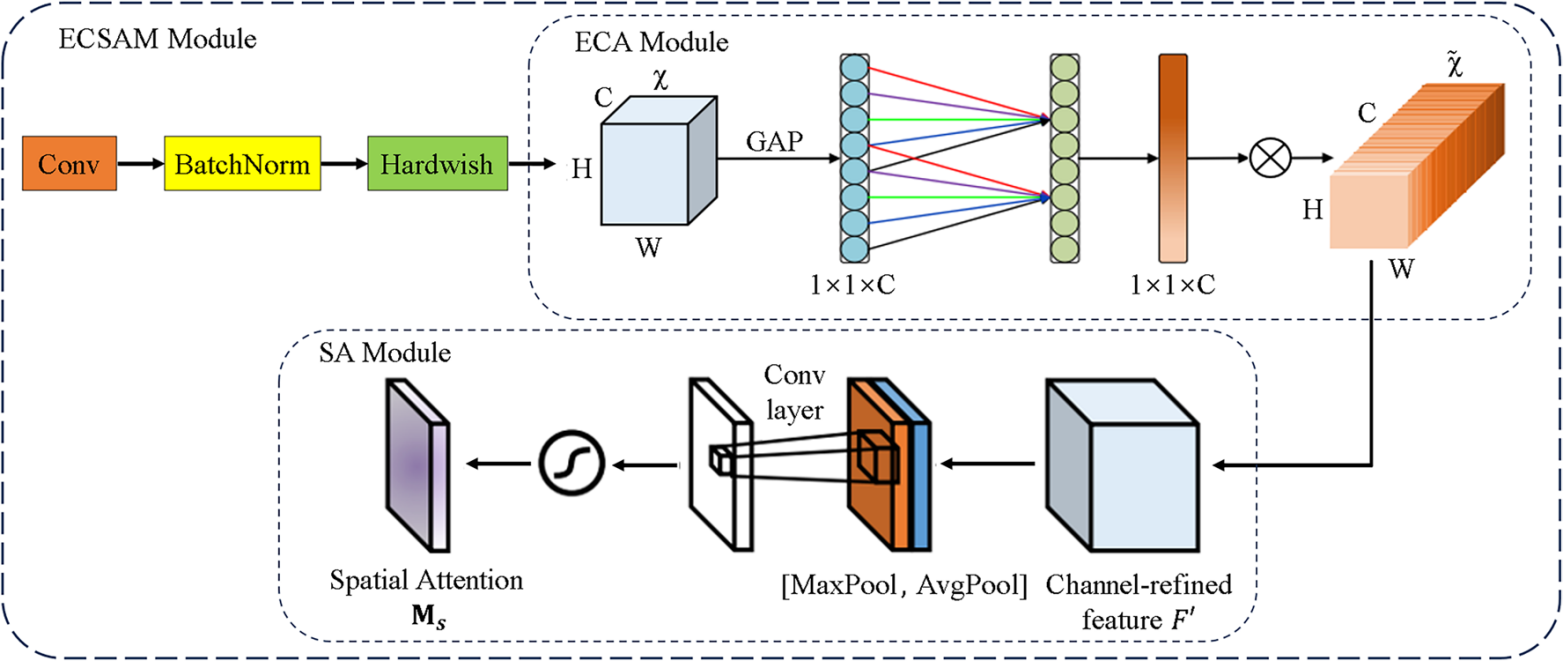
ECSAM module.

**Figure 12:**
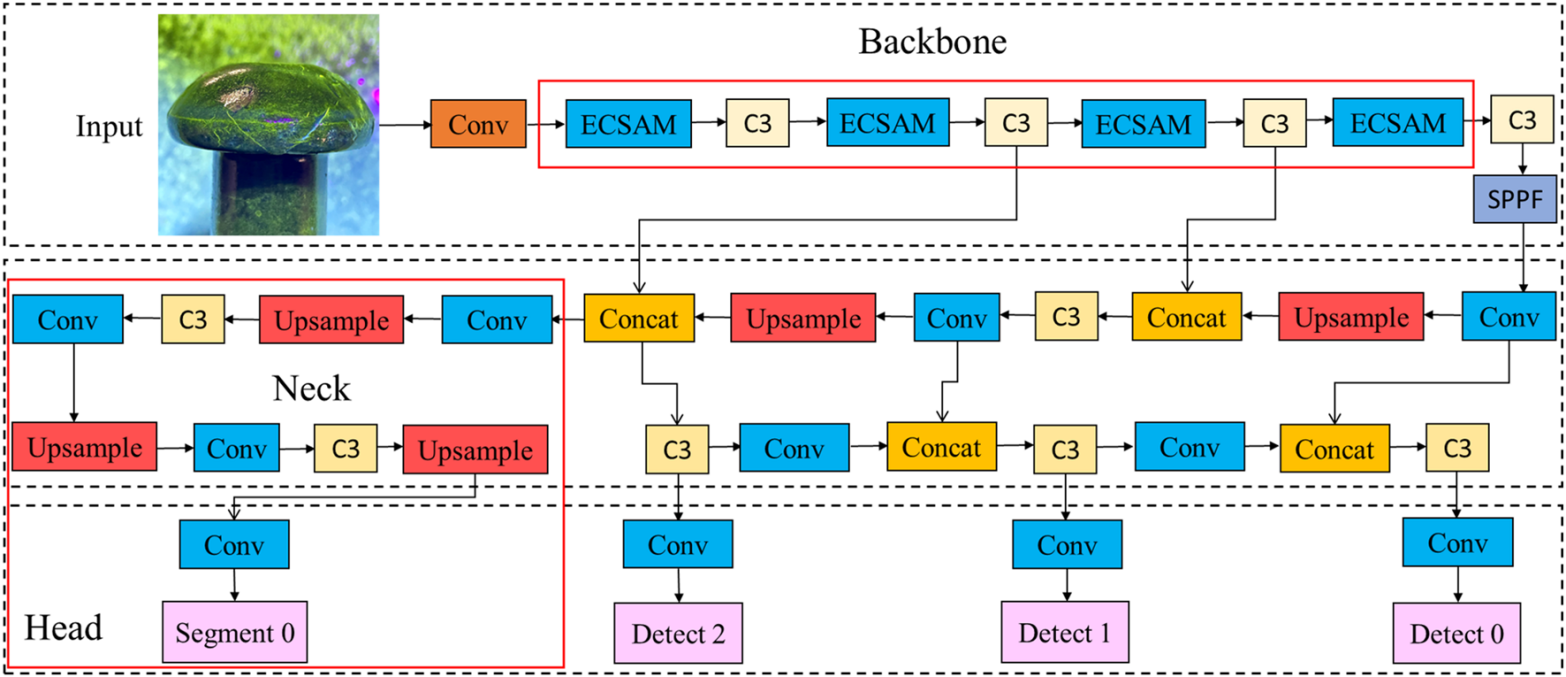
Improved overall modules.

In this chapter, a novel attention mechanism module ECSAM is proposed for fluorescent crack detection (Fig. 11). YOLOv5 uses a feature pyramid network to handle multi-scale objects. However, it still faces some challenges in detecting multi-scale fluorescent cracks. Therefore, ECSAM is inserted into the backbone of YOLOv5 to capture more discriminative features of the cracks, as shown in Fig. 12. ECSAM mainly consists of ECA (efficient channel attention)^20^ and spatial attention^21^. ECA is a lightweight channel attention mechanism that adapts the convolution kernel size to the number of channels and performs information exchange among neighboring channels. Spatial attention is a complementary module that focuses on the location of the cracks and reduces the interference from the background. Figure 11 illustrates the structure of ECSAM.

11 $$ECSAM = Conv\left( {BN\left( {Hardwish\left( {Concat\left( {ECA\left( X \right),SA\left( X \right)} \right)} \right)} \right)} \right)$$

12 $$ECA\left( X \right) = X \odot \sigma \left( {Conv_{k} \left( {AvgPool\left( X \right)} \right)} \right)$$

13 $$SA\left( X \right) = X \odot \left( {AvgPool\left( X \right) + MaxPool\left( X \right)} \right)$$

where $X$ is the input feature map with size H × W × C, $k$ is the adaptive kernel size, $\odot$ is element-wise multiplication, $\sigma$ is sigmoid activation and $+$ is element-wise addition.

The ECSAM module takes an input feature map with size H × W × C and performs the following sub-steps to enhance its channel and spatial attention:

(1) Apply a 1 × 1 convolution to the input feature map to reduce its dimensionality. (2) Perform batch normalization on the output feature map after convolution to stabilize the distribution of its values. (3) Use the Hardswish activation function to activate the normalized feature map and obtain the feature map χ. (4) Use the ECA module to compress the feature map χ along the spatial dimension and obtain a 1 × 1 × C vector that represents the channel-wise statistics of χ.(5) Use a one-dimensional convolution with an adaptive kernel size k to perform information exchange between each channel and its k nearest neighbors, and generate a new 1 × 1 × C vector that captures the channel-wise dependencies of χ.(6) Apply a sigmoid activation function to the new vector and obtain the interactive channel weights that reflect the importance of each channel in χ.(7) Multiply the feature map χ and the interactive channel weights element-wise and obtain the output feature map $\widetilde{\chi }$, which has enhanced channel attention. (8) Input the output feature map $\tilde{\chi }$ into the spatial attention module, which computes the average and maximum pooling along the channel axis and adds them together to obtain the spatial weight of $\tilde{\chi }$, which highlights the regions of interest in $\tilde{\chi }$. (9) Multiply the output feature map $\tilde{\chi }$ and the spatial weight element-wise and obtain the final output feature map of ECSAM module, which has enhanced spatial attention.

**Figure 13:**
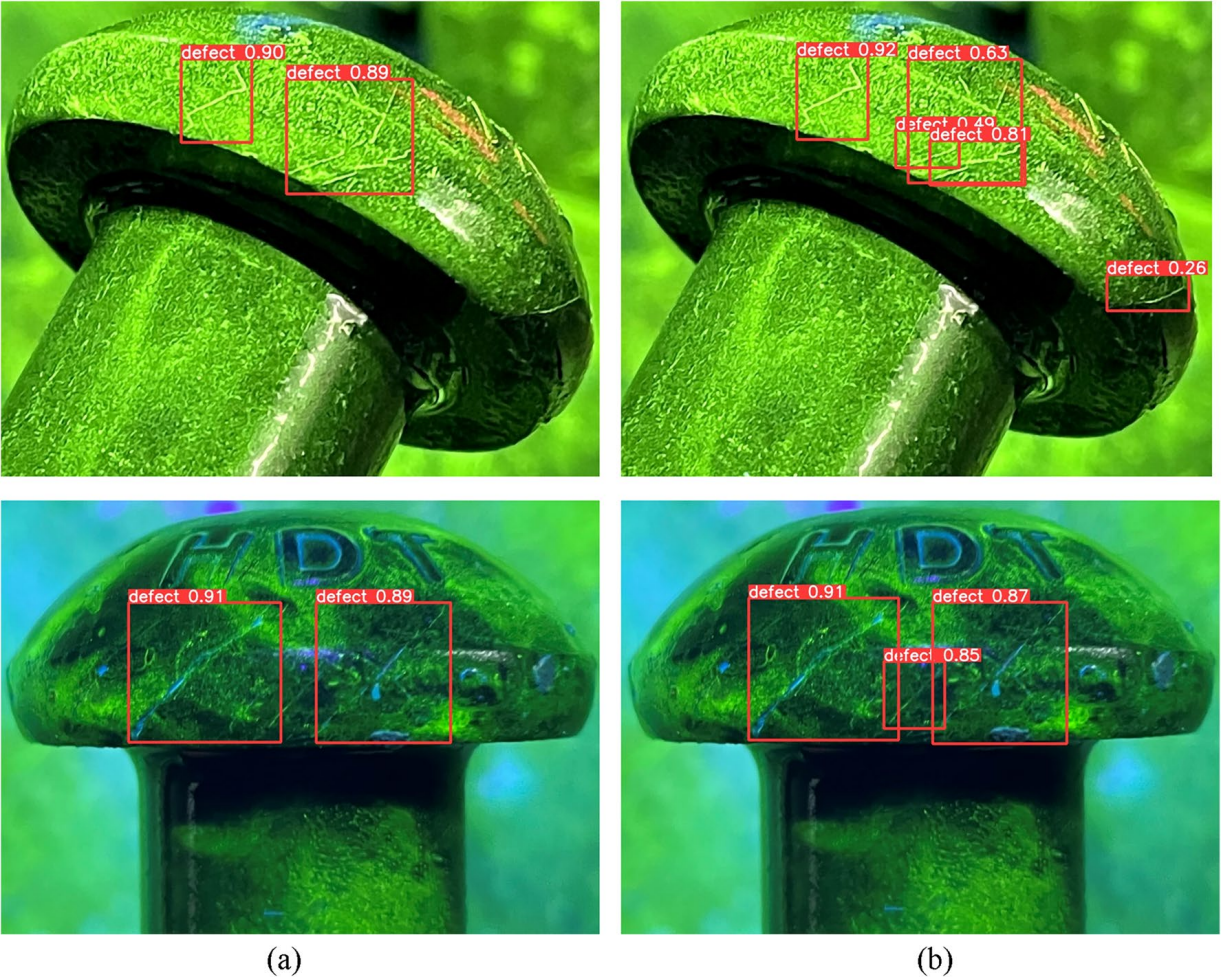
Improving detection examples of ECSAM with (**a**) YOLOv5, (**b**) YOLOv5 + ECSAM.

As shown in Table 3, experiments are conducted to compare ECSAM with different attention mechanisms in fluorescent crack detection tasks. The experimental results in Fig. 13 show that ECSAM can effectively improve the recall rate and mAP_0.5_ of multi-scale crack detection by enhancing the channel and spatial attention of the feature maps.

### Improvement of crack detection in uninstantiated regions based on multi task feature learning

In this section, improvement of crack detection in uninstantiated regions based on multi task feature learning is proposed. The samples of object detection annotation and semantic segmentation annotation are mixed together for training, and the two share the same feature extraction. Finally, the outputs of the two tasks are separated. This enables the model to learn crack space features while also supplementing the learning of more semantic features, improving its ability to detect cracks in difficult to instantiate areas. Then, the proposed fluorescent crack detection method is compared with semantic segmentation and it is argued that the proposed method can better capture the spatial information of the feature layer and handle the complex background of the fluorescent crack images.

Semantic segmentation algorithm such as deeplabv3+^22^ consists of an encoder-decoder structure. The encoder extracts high-level semantic features from the input image, and the decoder refines the features and produces a probability score for each pixel by category prediction. Deeplabv3+ can segment irregular cracks based on pixel classification, but it cannot distinguish different instances of cracks or reduce the interference of the fluorescent aggregation of the background. Therefore, if semantic segmentation is used to classify each pixel independently, the detection will be susceptible to interference from the fluorescence gathering, resulting in false detection.

**Figure 14:**
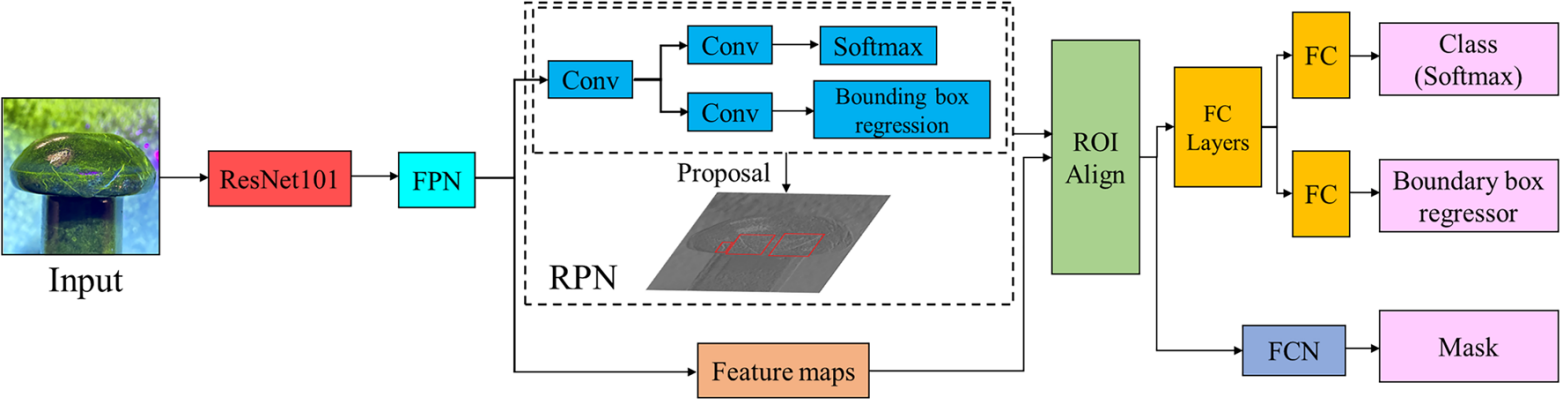
Architecture of Mask-RCNN for crack segmentation.

Instance segmentation algorithm such as Mask-RCNN^23^ builds on an object detection framework. As shown in Fig. 14, Mask-RCNN first extracts feature maps with different scales from the input image, then uses a region proposal network (RPN) to generate a series of candidate bounding boxes that overlap with the target to be detected. Next, it applies RoIAlign to crop and resize the features according to the size of the bounding boxes. Finally, it inputs the features into a mask branch for segmentation and a classification branch for category prediction. Mask-RCNN can not only segment uninstantiated cracks more precisely by adding a segmentation layer to the object detection algorithm, but also reduce the interference of the fluorescent aggregation of the background by focusing on the features that are positively correlated with the target.

**Figure 15:**
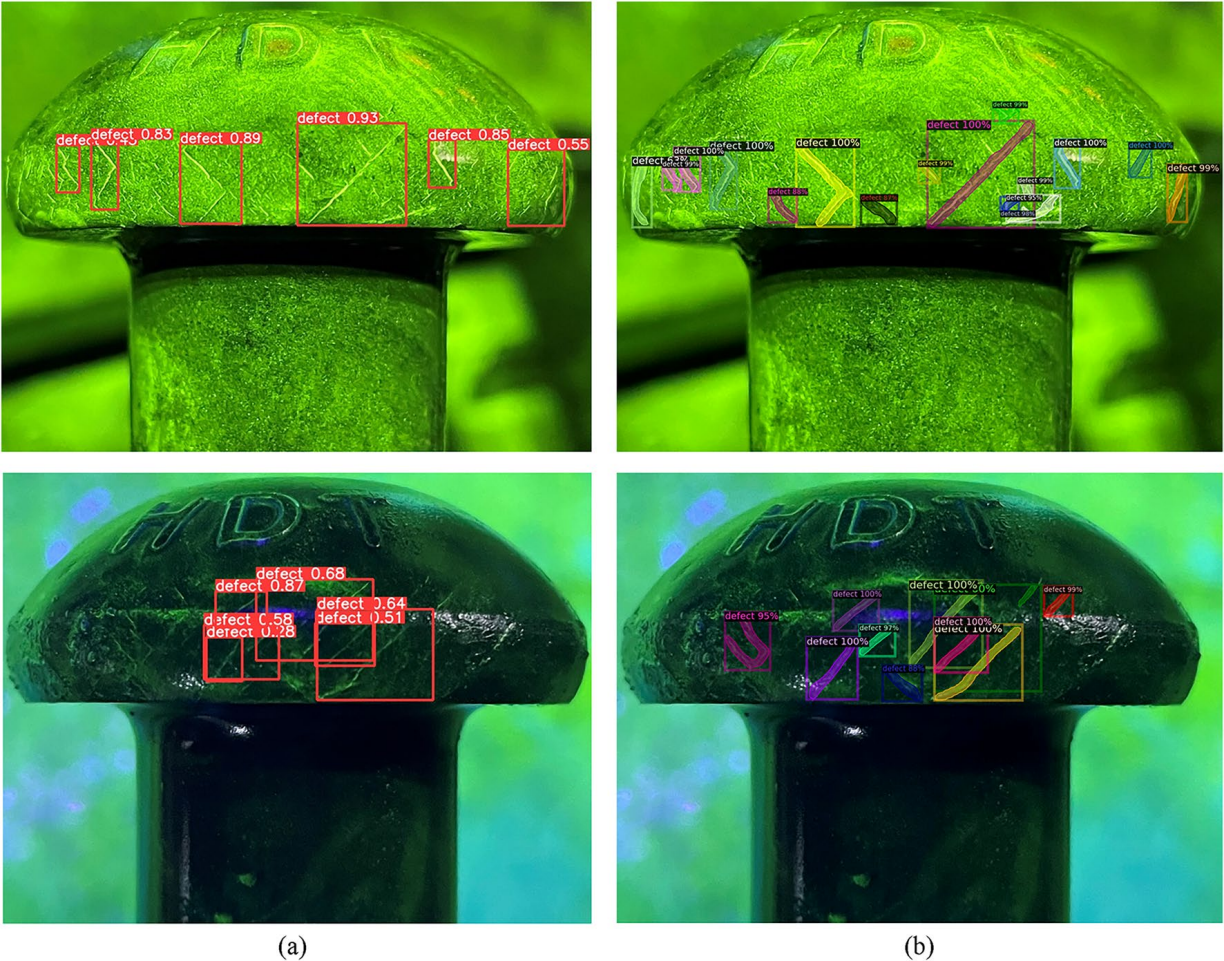
Detection examples of multi task feature learning method with (**a**) YOLOv5, (**b**) multi-task feature learning method.

As illustrated in Fig. 12, a segmentation prediction line is added to YOLOv5, which takes both segmentation and detection datasets as input. This allows the network to learn the characteristics of irregular cracks more fully and accurately^24^. This method has a similar principle to Mask-RCNN, but it has more fine-grained feature extraction and fusion, resulting in a higher mAP than Mask-RCNN. The effectiveness of the method is demonstrated by comparing the detection results of the baseline and the proposed method. As shown in Fig. 15, the results illustrate that the proposed method produce more detailed and accurate detection results of fluorescent cracks because it can integrate the semantic and spatial information of the dataset.

## Experiments results

In order to improve the detection indicator metrics of rivet fluorescent crack, YOLOv5x is used for subsequent experimental verification. YOLOv5x has the most layers of various types, which can extract more features and improve the detection accuracy and recall rate. Therefore, the experiments are based on YOLOv5x.

### Experimental setting

Experiments are conducted in this work to evaluate the performance of different models for fluorescent crack detection. All models are implemented using PyTorch and trained using NVIDIA GeForce RTX2080Ti. Due to the difficulty of obtaining defective rivet samples, 480 train rivet fluorescent crack images are collected as the dataset, which contain all the crack distribution situations of the rivets. In addition, online data augmentation is used in the ablation and comparison experiments, including random cropping, rotation, scaling, color and stitching transformations, which can increase the diversity of the data and improve the generalization ability of the model. Due to the small amount of data, no validation set is separately divided, and the data is split into a training set of 384 images and a test set of 96 images. Finally, the five-fold cross-validation method is used to test the indicators, and the final result is the average of the five test set results obtained in each iteration. The above methods all improved the generalization ability of the model, and reflected the effectiveness and reliability of the crack detection results obtained by the experiments in this paper.

### Experimental criterion

In this experiment, precision, recall, mAP, and mIoU (Mean Intersection Over Union) are used to evaluate the performance of the training model.

14 $$Precision = \frac{TP}{{TP + FP}}$$

15 $$Recall = \frac{TP}{{TP + FN}}$$

In the formula, TP (True Positive) represents the number of cracks predicted by the model that is consistent with the standard cracks. FP (False Positive) represents the number of prediction cracks that belong to the background. FN (False Negative) represents the number of real cracks predicted by the model as background. There are no true negatives (TN) in object detection^25^.

In the detection process, the coincidence degree $\alpha_{0}$ between the bounding box $BB$ and the ground truth $B_{gt}$ determines whether a bounding box is TP or FP. If the $\alpha_{0}$ is greater than the specified threshold, $BB$ is TP. Otherwise, it is FP. The threshold value $\alpha_{0}$ is set to 0.5 in this work. The coincidence degree $\alpha_{0}$ is defined as the formula:

16 $$\alpha_{0} = \frac{{area\left( {BB \cap B_{gt} } \right)}}{{area\left( {BB \cup B_{gt} } \right)}}$$

$BB \cap B_{gt}$ represents the intersection operation of the bounding box and the ground truth, $BB \cup B_{gt}$ represents the union operation of the two boxes. Area (∙) represents the total area of two boxes. Given the precision and recall, the precision and recall (PR) curve can be drawn. The area enclosed by the PR curve and the coordinate axis represents the detection precision of a single category, expressed by the average precision (AP), defined as the formula:

17 $$AP = \frac{1}{11}\mathop \sum \limits_{{r \in \left[ {0,0.1,0.2, \ldots .. , 1} \right]}} \mathop {{\text{max}}}\limits_{{ \tilde{r}:\tilde{r} \ge r}} p\left( {\tilde{r}} \right)$$

$p \left( {\tilde{r}} \right)$ represents the corresponding precision in the PR curve when the recall is $r$. Mean Average Precision of all categories mAP can represent the comprehensive object detection capability of the model, which is defined as the formula:

18 $$mAP = \frac{1}{{N_{cls} }}\mathop \sum \limits_{i} AP_{i}$$

where, $N_{cls}$ represents the total number of categories, $AP_{i}$ represents the average precision corresponding to each category $i$.

mIoU is the standard measurement of semantic segmentation. It calculates the intersection over the union of two sets: ground truth and bounding box. The calculation formula is as follows:

19 $$MIoU = \frac{1}{k + 1}\mathop \sum \limits_{i = 0}^{k} \frac{TP}{{FN + FP + TP}}$$

FPS (Frames Per Second) is an indicator of the speed of the algorithm in object detection, which reflects the time required by the algorithm to process one frame of image. The time for the detection algorithm to process one image includes the sum of the pre-processing, inference, and NMS time t, and its FPS is 1/t. The higher the FPS, the faster the algorithm and the higher the efficiency.

### Main results

In this section, the results of ablation experiments are presented to evaluate the effectiveness of each improvement method that proposed for fluorescent crack detection. YOLOv5x is used as the baseline model and different combinations of improvement methods are compared with it, as shown in Table 2. The results show that each improvement method can enhance the performance of the baseline model in terms of recall, precision, and mAP respectively. The decentralized target center and low overlap rate labeling method and Gaussian-weighted correction post-processing method can increase the recall rate by improving the model’s ability to detect dense cracks. ECSAM can strengthen the weight of key channels and spatial information in each feature map, and outperform CBAM and BiFPN^26^, which are existing attention mechanisms and weighted feature fusion. The multi task feature learning method can make full use of the semantic characteristics of cracks and boost the precision to 90.1%, which is higher than baseline. The best overall performance is achieved by integrating all improvement methods, which can reach 86.4%, 90.3%, and 61.1% for recall, mAP_0.5_, and mAP_0.5–0.95_ respectively, and surpass the baseline model significantly.

**Table 2:** Ablation experiment based on improved methods.

Model	Precision (%)	Recall (%)	mAP_0.5_ (%)	mAP_0.5–0.95_ (%)	FPS
YOLOv5	89.1	79.8	86.8	55.1	13.2
YOLOv5 + New Annotation (NA)	87.6	83.5	88.7	55.7	13.1
YOLOv5 + Soft-NMS	87.9	81.1	87.2	59.6	12.7
YOLOv5 + CBAM + BiFPN + NA	88.2	85.5	89.1	56.5	12.9
YOLOv5 + ECSAM + NA	88.4	85.7	89.6	56.7	13.0
YOLOv5 + ECSAM + Soft-NMS + NA	88.3	86.4	90.3	61.1	12.6
YOLOv5 + Segmentation head	90.1	81.5	88.4	55.4	12.8

**Figure 16:**
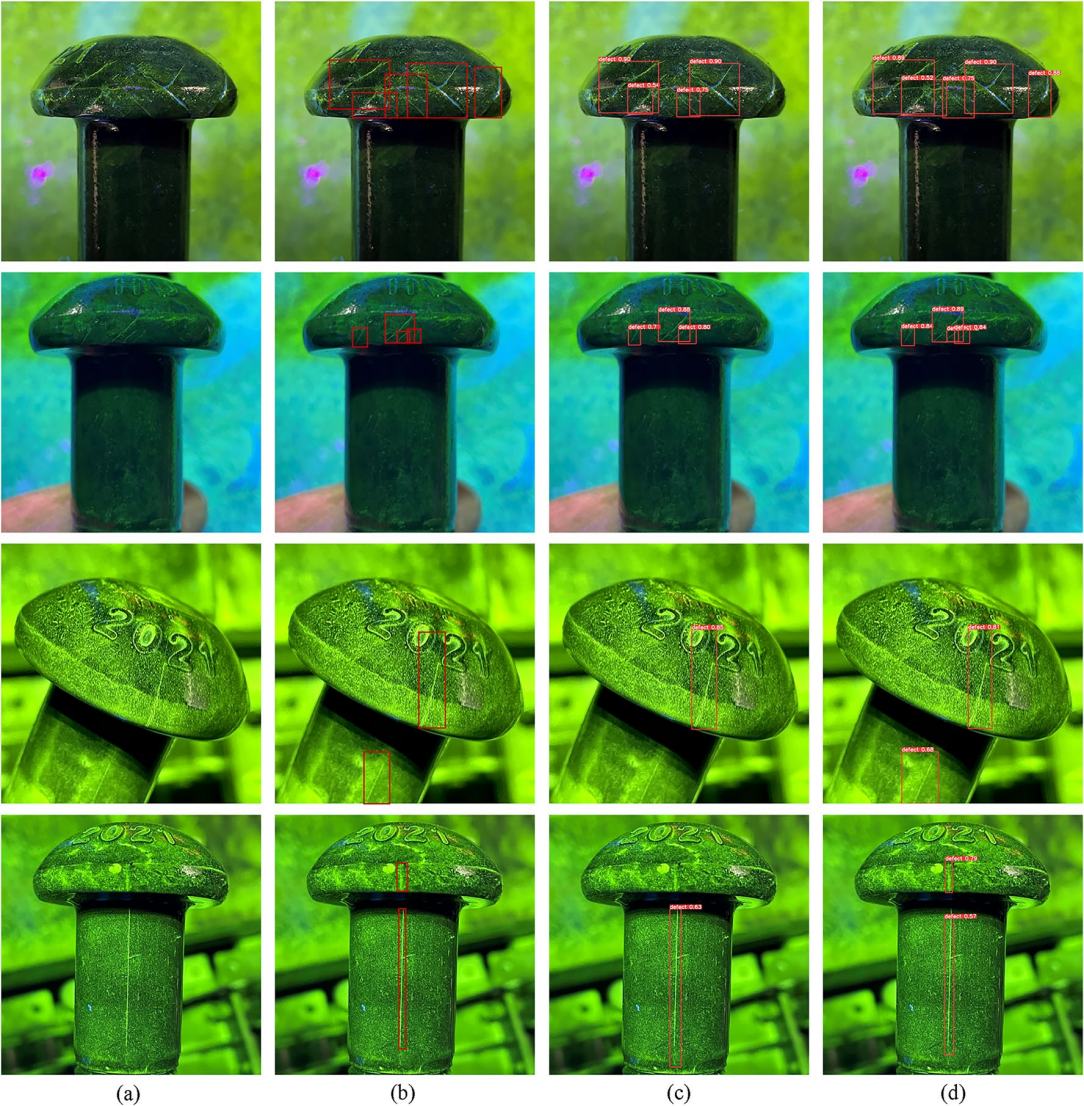
Comparison of detection results for baseline and our methods with (**a**) Images, (**b**) Ground truth, (**c**) Baseline, (**d**) Ours.

Some detection examples are shown in Fig. 16. Compared with the baseline, the best comprehensive group performs better in detecting dense, multi-scale, and irregular cracks.

Attention mechanisms are divided into channel, space, branch and temporal attention mechanisms^27^. Compared with channel, space and channel & space attention mechanisms, the effectiveness of the ECSAM module is verified. NAM in Table 3 is a channel attention mechanism based on the normalization, which is used in channel feature extraction. It can reduce the weight of less significant channel features and improve the accuracy of the model. SA is a common spatial attention mechanism that can weigh the feature map according to the importance of each spatial position. NAM + SA module is a nested attention module obtained by combining NAM and SA, which can achieve more efficient and flexible feature extraction. In addition, CA, SE and ECA are channel attention mechanism, while SimAM and CBAM are channel-spatial attention mechanism. The attention mechanisms used for comparison are added to the same position and number as ECSAM in the Backbone. ECSAM improves the recall rate by 5.9% compared to baseline while maintaining precision. This indicates that the ECSAM module can effectively capture the details and contextual information in the image, thereby enhancing the feature extraction ability of the Backbone.

**Table 3:** Performance comparison of ECSAM and other attention mechanisms.

Model	Precision (%)	Recall (%)	mAP_0.5_ (%)	FPS
YOLOv5	89.1	79.8	86.8	13.2
YOLOv5 + CA^28^	82.6	82.1	83.9	12.9
YOLOv5 + SE^29^	82.9	82.2	84.2	12.7
YOLOv5 + NAM^30^	89.0	81.7	87.2	13.2
YOLOv5 + SimAM^31^	78.8	83.1	85.5	13.2
YOLOv5 + CBAM^21^	85.6	85.5	87.6	13.1
YOLOv5 + NAM + SA	89.2	83.8	88.8	13.1
YOLOv5 + ECA^20^	84.8	84.0	87.3	13.1
YOLOv5 + ECSAM	88.4	85.7	89.6	13.0

The performance of the improved model is compared with other mainstream object detection algorithms, such as Faster-RCNN, YOLOX, YOLOv6, YOLOv7, and YOLOv8. As shown in Table 4, the improved model outperforms others, especially in recall rate. To ensure a fair comparison, the same settings are used for the model parameters, such as batch size and number of epochs. The proposed method reduces some speed of detection to achieve better results compared to the fastest YOLOv7 and YOLOv8, but still maintains a certain absolute speed. It has a higher performance in the recall rate and mAP_0.5_, which makes it suitable for the detection of rivet fluorescent cracks. The proposed method achieves a balance between speed and accuracy.

**Table 4:** Comparison between improved YOLOv5 and other mainstream object detection algorithms.

Model	Precision (%)	Recall	mAP_0.5_ (%)	FPS
YOLOv5-x	89.1	79.8	86.8	13.2
Faster-RCNN^32^	81.8	73.5	79.0	3.7
YOLOX-x^33^	85.5	80.6	85.7	12.8
YOLOv6-l6^34^	84.2	78.1	81.2	14.2
YOLOv7-x^35^	75.7	74.0	76.3	19.5
YOLOv8-x	88.2	84.1	89.7	20.6
YOLOv5x + ECSAM + NA + Soft-NMS	88.3	86.4	90.3	12.6

As shown in Table 5, the experiment also compares the mIoU of semantic and instance segmentation. Compared with deeplabv3+ and Mask-RCNN, YOLOv5 with segmentation line significantly improve the mIoU of fluorescent crack segmentation, which increased by 12% and 4%, respectively. Its mIoU is also slightly higher than UNet. This experiment proved the advantages and effectiveness of the multi task feature learning for fluorescent crack datasets.

**Table 5:** Comparison of crack segmentation performance.

Model	mIoU (%)
Deeplabv3+^22^	69.0
Mask-RCNN^23^	77.0
UNet^36^	79.6
YOLOv5 + Segmentation line	81.0

## Discussion

**Figure 17:**
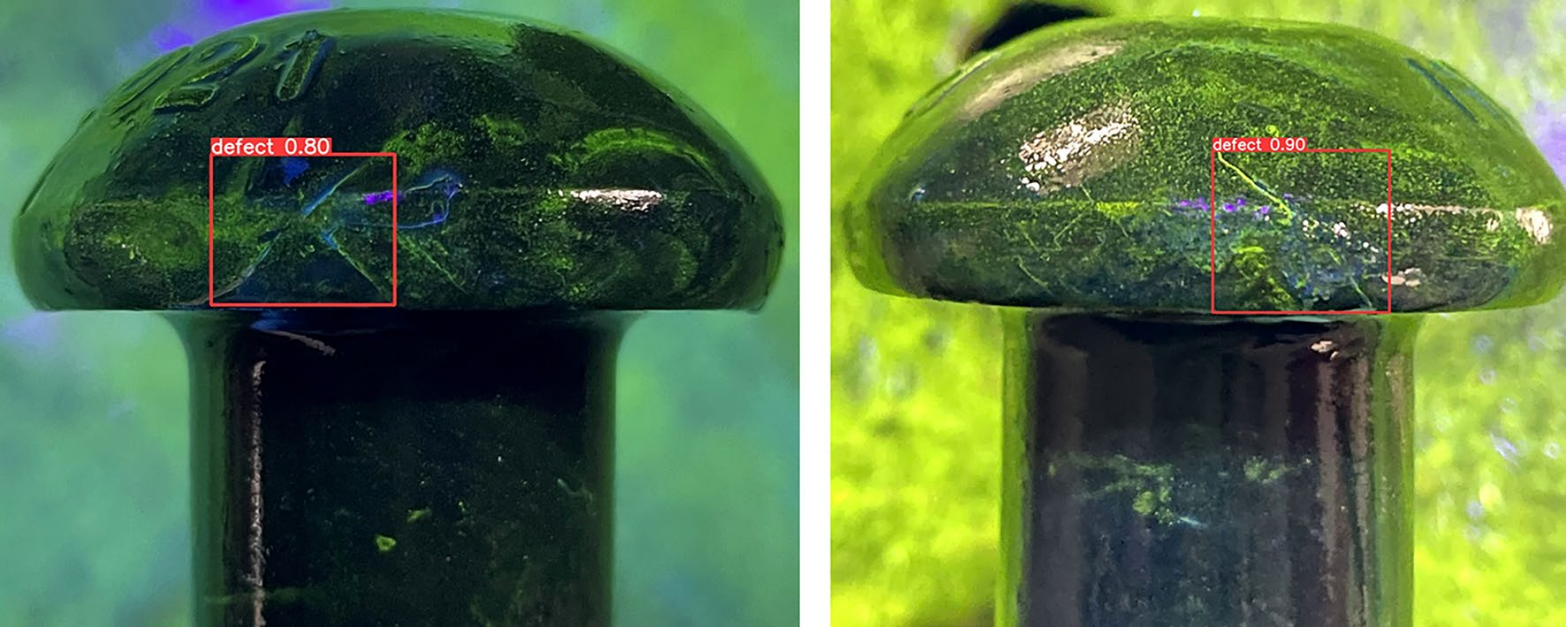
The results of detecting discontinuous cracks by decentralized target center and low overlap rate labeling method.

In general, the proposed pre-processing labeling method improves the recall rate of crack detection. However, cracks are usually discontinuous, and object detection algorithm may result in detecting fragmented cracks instead of single complete cracks. The decentralized target center and low overlap rate labeling method reduces or avoids the overlap between two annotation boxes and assigns cracks to different grids for prediction, while preserving the most important features of cracks and appropriately reducing the extraction of some less important features in dense crack regions. In addition, in the dataset, there are few cases of cracks with intermediate breaks, and most cracks are continuous. Therefore, in most cases, the annotation method can help to effectively detect complete cracks. Figure 17 shows the results of detecting discontinuous cracks by decentralized target center and low overlap rate labeling method. The negative impact of detecting fragmented cracks instead of single complete cracks on the metrics is much lower than the positive impact of the method on improving the recall rate of cracks in dense regions. This method shows better recall rate in high-density crack regions, and has advantages over the conventional edge-attached annotation method in terms of detection results and evaluation metrics. Furthermore, this method can be applied not only to dense crack detection, but also to other dense object detection tasks, such as overlapping packaged products on assembly line.

Moreover, the effectiveness of the proposed method for train rivet fluorescent crack detection is verified by experimental analysis. However, in some dense and uninstantiated crack regions, there are many tiny cracks distributed around some strip-shaped cracks, which may cause the crack prediction results to be inconsistent with the annotations. In practical detection, there may be situations where one annotation box contains multiple prediction boxes or one prediction box contains multiple annotation boxes, which affects the calculation of evaluation indicators and limits the further improvement of the indicators.

**Figure 18:**
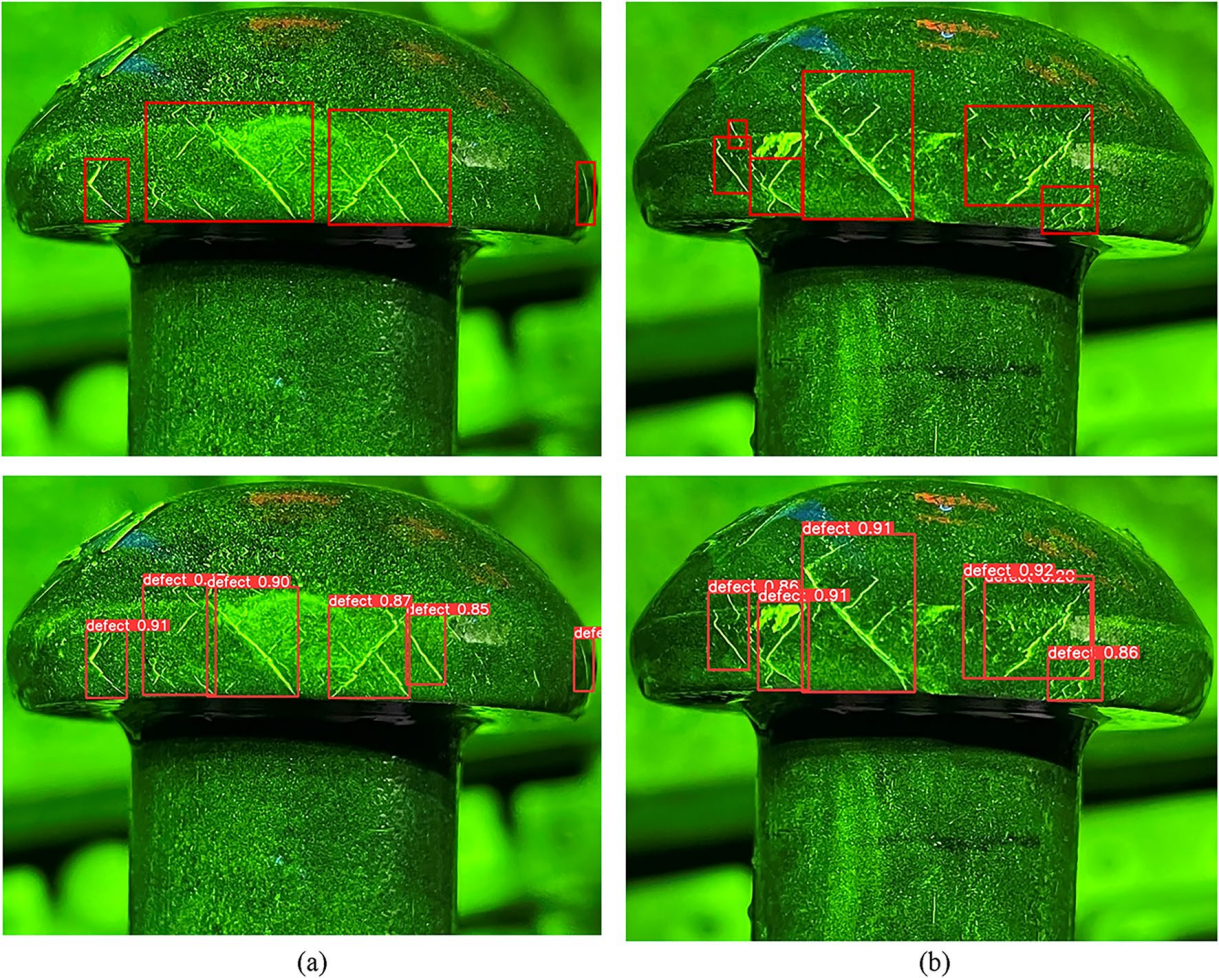
Analysis of existing detection results problems with (**a**) One annotation box contains multiple prediction boxes, (**b**) One prediction box contains multiple annotation boxes.

As shown in Fig. 18a, if a prediction box contains two annotation boxes, and the IoU of this prediction box and the two annotation boxes both exceed the set threshold, then the prediction box corresponds to the annotation box with the highest IoU when calculating the indicators. In this case, only one annotation box is correctly detected, and the other annotation box is missed, so the recall rate will decrease. As shown in Fig. 18b, if an annotation box contains two prediction boxes, and the IoU of this annotation box and the two prediction boxes both exceed the set threshold, then the annotation box corresponds to the prediction box with the highest IoU when calculating the indicators. In this case, only one prediction box is correctly detected, and the other prediction box is regarded as a false positive, so the precision will decrease. This also shows that the mAP is very sensitive to the number and position of the prediction boxes, and if the prediction boxes are too many or deviate too much from the annotation boxes, it will affect the value of mAP. Although some cracks may be detected in a new prediction combination form different from the annotation, the actual detection basically completes the requirement to detect all the surface cracks of the train rivet in the industrial production. In the future, the NMS post-processing or evaluation indicators of the detection algorithm can be optimized, so that the prediction boxes can more accurately regress to the annotations, and the detection results can be better reflected in the detection indicators.

## Conclusion

In order to meet the application requirements of intelligent detection of train rivet fluorescent magnetic particle inspection cracks, this paper carries out the research on the instance segmentation method for train rivet fluorescent magnetic particle inspection cracks. Focusing on the three problems of dense, multi-scale and uninstantiated cracks that affect the detection indicators, the detection ability of the model for the complex crack regions in the train rivet is improved, which effectively meets the application requirements of train rivet crack non-destructive testing. Meanwhile, based on the intelligent instance segmentation model proposed in this paper, relevant experiments are carried out to verify the feasibility and effectiveness of the improved algorithm. The recall rate and mAP_0.5_ of crack detection reached 86.4% and 90.3%, respectively.

The main contributions of this paper are summarized as follows:

1. A pre and post stage prediction box optimization method is proposed for crack detection in dense areas, aiming to address the problem of low recall rate of crack detection. The two stages are the decentralized target center and low overlap rate labeling method and the Gaussian-weighted correction post-processing method. The former method emphasizes the crack features of the non-overlapping part of the crack in the labeling, and assigns the cracks in the dense area to different grids for prediction, which enhances the accuracy of prediction box generation and regression, and increases the recall rate and mAP_0.5_ by 3.7% and 1.5%, respectively. The latter method lowers the confidence of overlapping prediction boxes, thus preserving more diversity of prediction boxes, and improves the recall rate by 1.3% and mAP_0.5–0.95_ by 4.5%.
2. An efficient spatial and channel attention mechanism (ECSAM) is proposed to address the problem in detecting some multi-scale cracks. The ECSAM attention mechanism can enhance the channel and spatial annotation features of cracks in the feature map, improve the model’s attention ability to cracks, and increase the recall rate by 5.9% compared with the baseline.
3. A multi-task crack feature learning method is proposed, aiming to alleviate the problem of detecting fuzzy boundary and uninstantiated cracks. The multi-task learning utilizes the semantic and spatial features of fluorescent cracks, enabling the network to obtain more comprehensive crack information from both detection and segmentation datasets, thus improving the indicators of crack detection and reducing the background interference.
4. A single coil non-contact train rivet composite magnetization device is built and a rivet fluorescent magnetic particle inspection crack dataset is constructed. In order to solve the problems of large volume of existing industrial equipment and rivet surface being easily burned by current. The device can detect the radial and axial cracks of rivets at the same time. In addition, the effectiveness of the device is verified by magnetic sensitive test piece experiment. The device has good versatility and is not sensitive to the shape of rivets.

## Data Availability

The datasets analyzed during the current study available from the corresponding author on reasonable request.
